# Choreographing Oscillatory Hydrodynamics with DNA-Coated Gold Nanoparticles

**DOI:** 10.1021/jacs.4c06868

**Published:** 2024-06-28

**Authors:** Anish Rao, Ana Sánchez Iglesias, Marek Grzelczak

**Affiliations:** † Centro de Física de Materiales CSIC-UPV/EHU, Paseo Manuel de Lardizabal 5, 20018 Donostia San-Sebastián, Spain; ‡ Donostia International Physics Center (DIPC), Paseo Manuel de Lardizabal 4, 20018 Donostia-San Sebastián, Spain

## Abstract

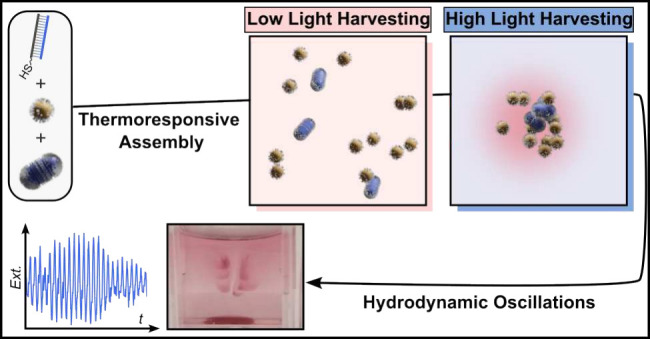

Periodic responses to nonperiodic energy inputs, such as oscillations, are hallmarks of living systems. Nanoparticle-based systems have largely remained unexplored in the generation of oscillatory features. Here, we demonstrate a nanosystem featuring hierarchical response to light, where thermoplasmonic effects and reversible DNA-hybridization generate thermal convective forces and ultimately, oscillatory hydrodynamic flows. The slow aggregation of gold nanoparticles (AuNPs) serves as a positive feedback, while fast photothermal disassembly acts as negative feedback. These asymmetric feedback loops, combined with thermal hysteresis for time-delay, are essential ingredients for orchestrating an oscillating response.

Successful preparation of self-assemblies has long been recognized as a significant achievement.^1−3^ Researchers have devoted considerable efforts to understanding and mimicking natural self-assembled structures and functions to systems composed of nonbiological components.^4^ This has rendered remarkable developments in regulating interparticle interactions to form static^1,2,5^ and dynamic self-assemblies.^3,6,7^ Dynamic assemblies can, under suitable stimuli, exhibit switchable,^8−10^ transient,^11^ or oscillating^12^ behaviors. Particularly attractive are systems demonstrating periodic responses under nonperiodic energy supply, i.e., *self-oscillations*(13) since they have been envisaged to outperform their steady-state counterparts, especially for catalytic applications.^14^ Despite recent advancements,^12,15−20^ designing functional and modular self-oscillators presents a formidable challenge.

Traditionally, chemical oscillations were achieved using organic and inorganic reagents in continuous stirred tank reactors and flows^21,22^ or enzymatic networks.^23^ The key design components involve *positive* and *negative* feedback loops separated by a *time delay*.^24^ Although the above-mentioned oscillations have been exploited to induce similar patterns in colloids,^25^ polymers,^26^ or gels,^27^ macroscopic manifestation of oscillatory patterns, detectable by the naked eye, seems to be reserved only for molecular systems. The central hypothesis of the present work is that a hierarchical design of NP-based systems, where thermoresponsive surface ligands regulate the reversible clustering of NPs under uninterrupted light, can eventually generate strong thermal convection forces that eventually result in hydrodynamic oscillations (Figure 1).

**Figure 1:**
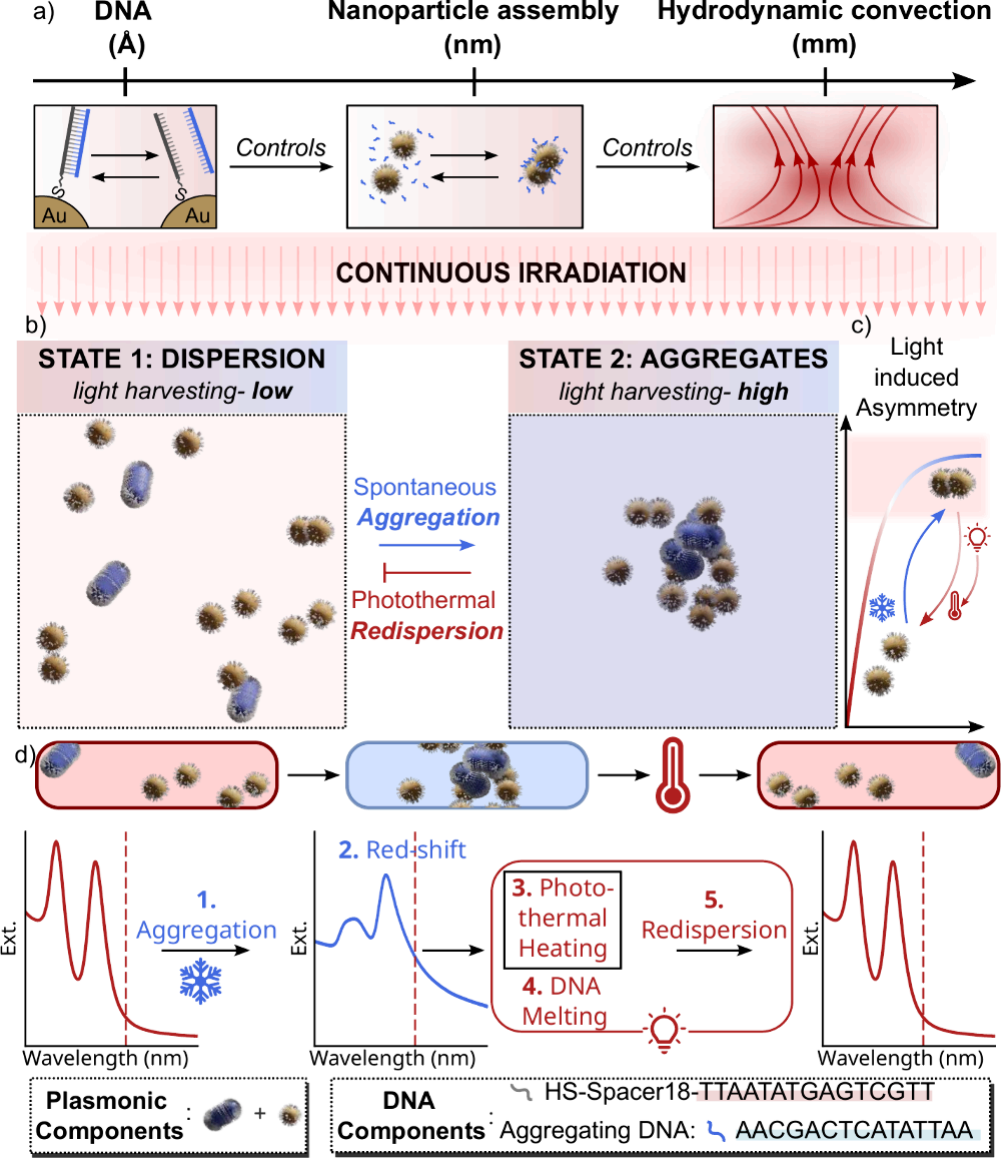
a) Thermosensitive hybridization of DNA-based ligands controls the reversible assembly of nanoparticles, setting conditions for oscillatory convection at the macro scale. b) Feedback mechanisms: A binary mixture of AuSs and AuNRs spontaneously assembles through DNA hybridization (positive feedback). Once assembled, the system absorbs enough NIR light to trigger photothermal disassembly (negative feedback). c) Oscillatory features arise from light-induced asymmetry, accelerating disassembly. d) Schematics showing the sequence of processes occurring in our system.

We selected DNA-triggered spontaneous and nonspecific aggregation of AuNPs as *positive feedback* and heat-induced redispersion as *negative feedback* (Figure 1). Here, aggregation-induced redshift in the extinction of AuNPs increases the light-harvesting properties of the system in the assembled state under nonperiodic irradiation with a laser. This increased light absorption produces photothermal-heating that results in melting of DNA and ultimately, the disassembly of AuNPs (*light-induced asymmetry*, see Figure 1c). The *time delay* was introduced through thermal hysteresis which is a typical feature of self-assembling NP.^28,29^ Under suitable experimental conditions (temperature and laser power), we observed that local heating by AuNPs, coupled with thermoresponsive self-assembly could induce forces (*thermal buoyancy*)^30^ that resulted in hydrodynamic oscillations. Our system thus exhibits hierarchical features, where, angstrom scale molecular components control the assembly of NPs, which finally translates into millimeter-scale oscillating hydrodynamic flows (Figure 1a).

To ensure the increase of extinction at ∼808 nm (resonant with laser) we selected a mixture of DNA-coated gold nanospheres (AuSs, diameter = 28 ± 2 nm, Figure S1) and gold nanorods (AuNRs, length = 54 ± 4 nm, width = 18 ± 1 nm, Figures S2 and 1). For more details on synthesis and ligand exchange, see Sections S1.2 and S1.3. These DNA-coated AuNPs aggregated in the presence of complementary DNA (aggregating DNA) under high ionic strength (∼200 mM NaCl) due to the *salting-out* effect (Figure 2b).^31^ Note that unary systems of AuSs or AuNRs failed to exhibit the desired spectral shifts upon aggregation. Although AuSs exhibits redshifts during aggregation, their photothermal-heating is relatively poor (weak negative feedback) (see Figures S4 and S5).^32^ Conversely, AuNRs despite having high photothermal-heating abilities, often display minor redshifts due to more stable side-to-side assembly (see Figure S6).^5^ By mixing and assembling AuNRs and AuSs, unstructured aggregates form that exhibit consistent redshifts along with high photothermal-heating abilities, thereby fulfilling both criteria for choreographing an oscillating response.

**Figure 2:**
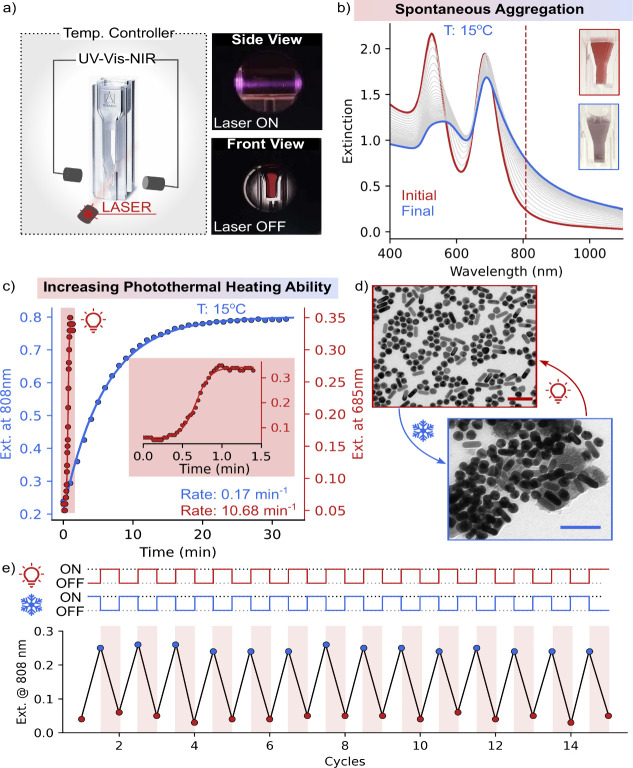
a) Schematic of experimental setup with CW laser at 808 nm orthogonal to UV–vis–NIR probe. Optical images showing both the front and side views of the cuvette containing AuNPs in the temperature controller. The side view shows the path of the laser during the irradiation. b) Time-dependent UV–vis–NIR spectra of AuNP mixture during assembly showing consistent redshifts and simultaneous decrease in the extinction intensity. Inset shows optical images of initial and final states. c) Increase in extinction intensity at 808 nm during the course of aggregation and change in extinction at 685 nm upon photothermal redispersion. d) TEM images of the disassembled and assembled nanoparticles. Scale bar = 100 nm. e) 15 cycles showing temperature-induced assembly (blue), and light-induced disassembly (red).

Our system is designed to respond to external stimuli only in the assembled state (negative feedback), absorbing NIR laser energy (808 nm) exclusively upon assembly (see Figure S3).^33^ This laser wavelength is detuned from the plasmonic responses of both components (∼523 nm for AuSs, ∼ 685 nm for AuNRs) in their dispersed state, thereby minimizing their interaction with the stimulus. Only upon the assembly of NP mixture, a redshift occurs, increasing the absorbance at 808 nm (Figure 2b, c). The rate of aggregation was estimated to be 0.17 min^–1^. The unstructured nature of the assembly is further evident in the transmission electron microscopy images shown in Figure 2d. Upon aggregation, the extinction at the laser wavelength progressively increases, triggering the disassembly within 2 min (red curve in Figure 2c). The rate of redispersion was 10.68 min^–1^. The assembly disassembly cycles were performed 15 times by decreasing the temperature to 15 °C, while disassembly was initiated using laser power of 1.9 W (Figures 2e and S7).

A delay in the system’s response to external stimulus is critical for obtaining oscillations, which allows for avoiding monotonous steady states.^24^ Although different strategies can be employed to introduce a time-delay, hysteresis is one attractive means.^24^ To evaluate the presence of thermal hysteresis, we conducted reversible assembly under a temperature-ramp of 0.5 °C/min, spanning from 10 to 40 °C (Figure 3a). We observed a freezing temperature (*T*_f_) of 14.4 °C and a melting temperature (*T*_m_) of 22.4 °C under dark conditions. Interestingly, these temperatures shifted under laser irradiation. Specifically, as laser power increased, both the freezing and melting temperatures decreased (see Figure 3b). Although these shifts are small, analysis performed by following extinction at 523 nm (corresponding to AuSs) shows a similar trend of decreasing transition temperatures. These shifts are attributed to the photothermal-heat generated by the NPs under laser irradiation. With the presence of additional photothermal-heat, more amount of heat needs to be *taken away* from the system to assemble the NPs, thereby decreasing the freezing temperature. Conversely, less heat needs to be *supplied* to disassemble the NPs, leading to a decrease in the melting temperature as well (see Figure S8). Note that at laser power of ∼135 mW, the system failed to assemble even at 10 °C, due to the excessive photothermal-heating, emphasizing the critical role of laser power in modulating assembly disassembly dynamics.

**Figure 3:**
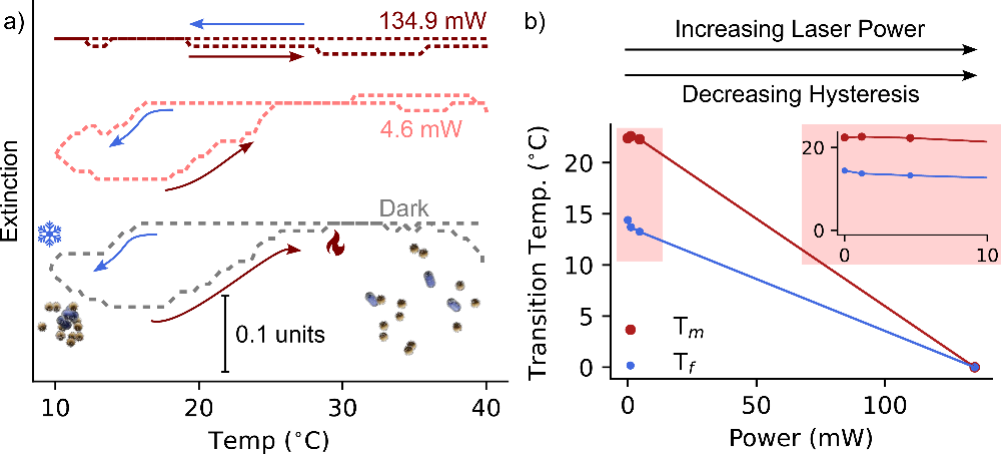
a) Variation in the hysteresis of the system with increasing laser powers. The thermal hysteresis was followed using extinction at ∼685 nm at a scan rate of 0.5 °C/min. b) Schematic of the effect of increasing laser power on hysteresis. Graph shows the variation of transition temperatures during heating (*T*_m_, red) and cooling (T_f_, blue) versus laser power. The inset shows the variations in transition temperatures at low laser powers.

With all the feedback mechanisms in place, we investigated the conditions for observing the onset of oscillations. As an initial state, we set a completely precipitated AuNP mixture on the bottom of the cuvette. The solution was irradiated with a CW 808 nm laser a few mm above the precipitates and UV–vis–NIR was recorded orthogonal to the laser (Figure 2a). The spectra were recorded at intervals of 2 s for 2 h. Under low laser power (∼4.6 mW), NPs remained aggregated due to insufficient photothermal-heating, resulting in unchanged extinction at LSPR (Figure 4a, black line). Under high laser power (∼562.5 mW), we observed monotonic redispersion of NPs (Figure 4a, red line). Intriguingly, at laser powers adequate for redispersion, but overwhelming the aggregation of AuNPs (∼134.9 mW), the extinction value at 685 nm exhibited oscillations (Figure 4a, blue line). The oscillating response was further analyzed using Fourier analysis, revealing the major component of oscillation corresponding to a period of 0.028 Hz, or a frequency of ∼36 s (Figure 4b,c).

**Figure 4:**
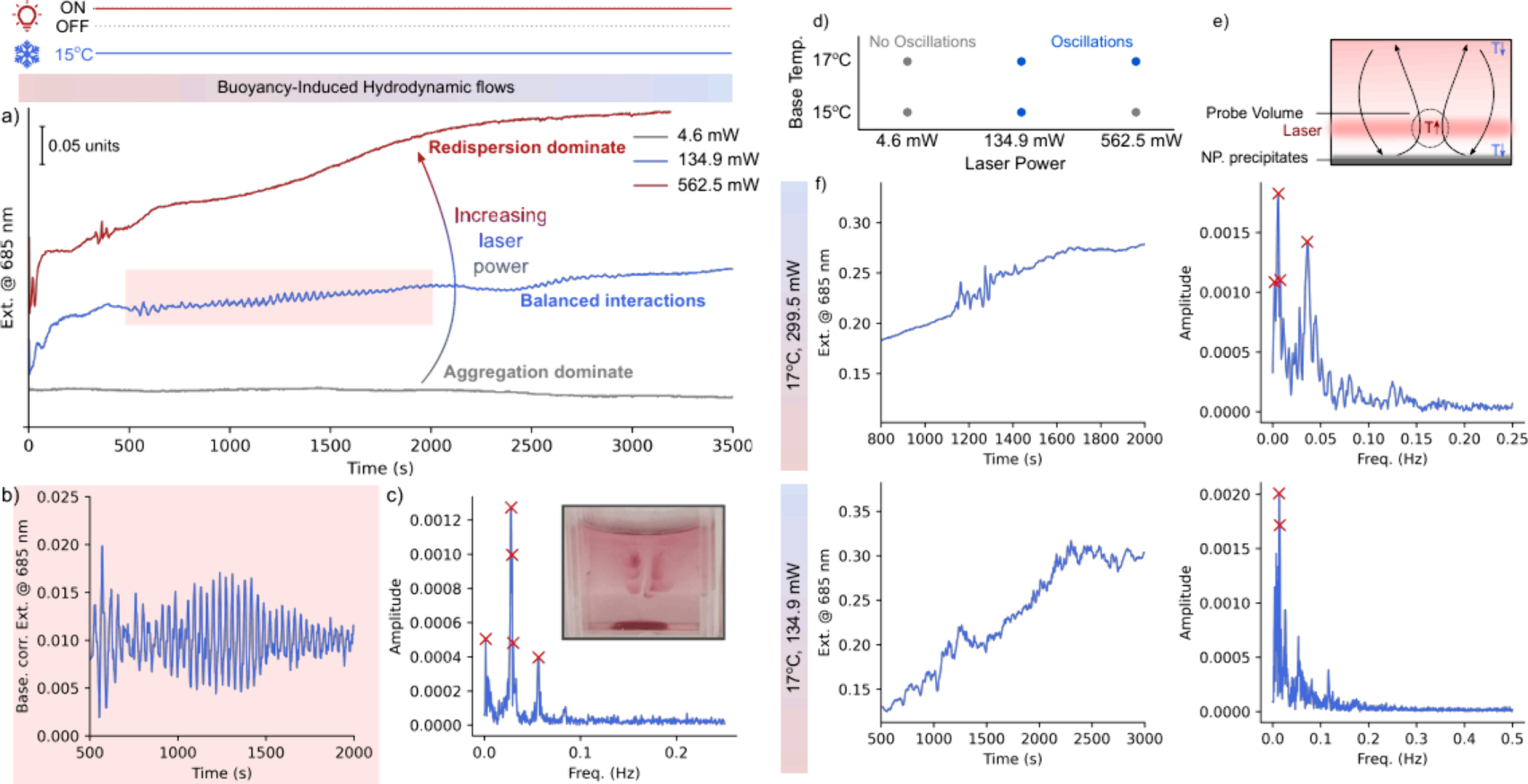
a) Extinction response of the system (15 °C, ∼134.9 mW) showing oscillatory signature. b,c) Zoomed UV–vis–NIR trace (500–1000s, highlighted in pink) and corresponding Fourier analysis. Inset in c) digital image showing a colored wave-like pattern. d) Phase space of base temperature (aggregation) and laser power (redispersion) with oscillatory conditions in blue. e) Schematic of hydrodynamic oscillations from temperature-induced flows. f) UV–vis–NIR and corresponding Fourier analysis of the system at 17 °C and a laser power of ∼299.5 and ∼134.9 mW.

Oscillations were also observed under alternative conditions, such as at 17 °C at ∼134.9 and ∼299.5 mW (see Figure 4d). Interestingly, we observed increasing periods with increasing laser power, with the major oscillating frequency increasing from ∼71 s (0.014 Hz) to ∼166 s (0.006 Hz) while increasing the laser power from ∼134.9 to ∼299.5 mW at a base temperature of 17 °C.

We postulate that the oscillations originate from temperature-dependent changes in the buoyancy of the dispersion, leading to organized convective flows. We observed oscillations in the extinction intensity at ∼523 nm corresponding to the extinction of AuSs (see Figure S9). This observation hints at the possibility of oscillations originating from changing amounts of NPs in the UV–vis–NIR probe volume. Notably, we observed a colored wave-like pattern in the solution, providing additional evidence for the hydrodynamic origins of the oscillations (optical image in Figure 4c). This pattern is produced by the convective motion of the solvent dragging with itself, the NP mixture. Similar flow-based oscillations have been shown in the literature,^34^ but they require higher sample volume and a need for a volatile solvent to establish a strong enough temperature gradients. The distinct benefit of using plasmonic NPs is their ability to establish potent enough temperature gradients even at 200 μL solutions.^34^

To induce oscillations, the following critical parameters were established: temperature gradient, thermoresponsiveness, and feedback loop asymmetry. We confirmed these parameters through a series of negative control experiments which led to stationary, nonoscillatory responses. First, no oscillation was observed for CTAB-coated AuNRs (see Figure S11). Second, the use of either AuSs or AuNRs generated a stationary state that was attributed to poor photothermal-heating (in the case of AuSs) (Figure S4), or negligible redshifts in the extinction spectrum upon assembly (in the case of AuNRs) (Figure S6). Third, 670 nm laser failed to generate oscillatory dynamics due to its inability to induce asymmetry in the system since both unassembled and assembled states possess similar extinction. These experiments underscore and reiterate the critical need for thermoresponsive assembly, as well as the significant increase in extinction at the laser illumination during the assembly, as essential components for obtaining the oscillating response. Finally, we ruled out the contribution of reversible assembly/disassembly of NPs to oscillatory features. LSPR shift is a signature of decreased interparticle distance that increases plasmon coupling and thereby controls the photothermal effect. In the present case, the LSPR shifts at the λ_*max*_ were negligible throughout the oscillations (see Figure S10).

Typically, oscillations by nature are dynamic, nonlinear and in our case, sensitive to the initial conditions as well.^35−40^ We performed multiple control experiments ranging from a completely unassembled state (see Figure S12) to aggregated dispersion of AuNPs (see Figure S13), observing hydrodynamic oscillations only when starting from completely precipitated dispersion of AuNPs.

Our study shows the principles for developing NP-based oscillators, and extends beyond the conventional approach of regarding self-assemblies as static end products. We employ DNA-coated AuNPs that exhibit temperature-dependent self-assembly and photothermal-heating to induce an oscillating response. Our design ensures that the self-assembled state preferentially interacts with the laser, thereby showing asymmetry, which is recognized as a crucial feature in nonequilibrium systems. Our study positions AuNPs as compelling and attractive models for studying nonequilibrium systems and realizing properties under such conditions.

## Supporting Information Available

Synthesis details and ligand exchange protocol for AuNRs and AuSs; schematic demonstrating the experimental setup; UV–vis–NIR, TEM measurements for AuSs and AuNR, and control experiments (PDF)
