# Supplementary material for: Choreographing Oscillatory Hydrodynamics with DNA-Coated Gold Nanoparticles
Source: J Am Chem Soc. 2024 Jun 28;146(27):18236–40. doi: 10.1021/jacs.4c06868 (PMC11240255; doi:10.1021/jacs.4c06868)
Supplement: Supplementary file 1 — ja4c06868_si_001.pdf [file ja4c06868_si_001.pdf]

Supporting Information for Choreographing Oscillatory Hydrodynamics with DNA-Coated Gold Nanoparticles

Anish Rao,[*,†] Ana Sánchez Iglesias,[†] and Marek Grzelczak[*,†,‡]

†*Centro de Física de Materiales CSIC-UPV/EHU, Paseo Manuel de Lardizabal 5, 20018 Donostia San-Sebastián, Spain*

‡*Donostia International Physics Center (DIPC), Paseo Manuel de Lardizabal 4, 20018 Donostia-San Sebastián, Spain*

E-mail: anish.rao@ehu.eus; marek.g@csic.es

S1 Materials and Methods

S1.1 Chemicals

Gold (III) chloride trihydrate ($HAuCl_4 \cdot 3\,H_2O$, ≥99.9%), hexadecyltrimethylammonium bromide (CTAB, ≥99%), sodium borohydride ($NaBH_4$, 99%), hydroquinone (HQ, ≥ 99%), silver nitrate ($AgNO_3$, ≥99.0%), poly (sodium 4-styrenesulfonate) (Na-PSS, M_w=70000 g/mol), hydrochloric acid (HCl, 37%), sodium citrate tribasic dihydrate (≥ 99%), sodium chloride, phosphate buffer, sodium dodecyl sulfate, RNA-free BioPerformance certified water were purchased from Aldrich-Merck. All chemicals were used without further purification. DNA sequences were purchased from Biomers. Milli-Q grade water (resistivity 18.2 MΩ cm at 25°C) was used in all experiments. All glassware and stirrer bars were cleaned with aqua regia, rinsed with Milli-Q water, and dried before use.

S1.2 Synthesis and ligand exchange of AuSs

AuSs were synthesized using a reported seeded growth method.[1] First, Au seeds were synthesized by heating a solution of trisodium citrate (150 mL, 2.2 mM) for 15 min under vigorous stirring until boiling. Subsequently, 1mL of $HAuCl_4$ (25 mM) was added to the solution in one shot. The colour of the solution changed from yellow to bluish-grey and finally to light pink in 10 min. Following the synthesis of seeds, cyclic addition of metal precursor and removal of NPs was performed first by cooling the solution to 90°C and then adding $HAuCl_4$ (1 mL, 25 mM) in two steps 30 min apart. After an additional 30 min, a portion of the growth solution (55 mL) was removed and the remaining mixture (98 mL) was mixed with water (53 mL) and sodium citrate (2 mL, 60 mM). This process was repeated 2 times to obtain spherical gold nanoparticles (AuSs) with a diameter of ∼28 nm.

Ligand Exchange: DNA functionalization (15-mer) of AuSs was carried out using the reported literature protocol.[2] For DNA functionalization, the redispersion DNA sequence was modified at the 5'-end with a thiol group followed by a polyethylene-glycol spacer followed by the nucleotide sequence (SH-Spacer18-nucleotide sequence). The complete list of DNA sequences is provided in Table S1. An excess of thiolated oligonucleotides (0.1 mM) was added to AuSs@Citrate (5.55 mL) containing sodium dodecyl sulfate (SDS) 0.1% and phosphate buffer 0.01 M, followed by incubation at room temperature for 20 min. To improve oligonucleotide binding, a salt-ageing process was carried out by sequential addition of 25, 25, 75, 125 and 250 μL of a solution containing NaCl 2 M, SDS 0.01% and phosphate buffer 0.01 M, up to a final concentration of NaCl 0.2 M. Each salt aging step was sonicated (10 s) and incubated (20 min), and the last one was incubated for 12 h, at 25°C. To remove the excess oligonucleotides, the solutions were centrifuged (7000 rpm, 30 min) and re-dispersed in 2 mL of SDS 0.01%. This step was repeated three more times.

Table S1: Table showing the DNA sequences used in the study.

Name	Oligonucleotide Sequence
Thiolated DNA	SH-Spacer18-TTA-ATA-TGA-GTC-GTT
Aggregating DNA	AAC-GAC-TCA-TAT-TAA

S1.3 Synthesis and ligand exchange of AuNRs

Au Seeds: The gold seeds were prepared by reduction of $HAuCl_4$ with $NaBH_4$ in the presence of a cationic surfactant (CTAB) in a 20 mL scintillation vial. To an aqueous mixture containing CTAB (100 mM, 4.7 mL) and $HAuCl_4$ (50 mM, 0.025 mL), freshly prepared ceNaBH4 (10 mM, 0.3 mL) was added under vigorous stirring at room temperature. The solution turned from yellow to brownish immediately and the stirring was stopped after 2 min. The seed solution was aged at room temperature for 30 min before use to promote the decomposition of excess sodium borohydride.

AuNR growth: Gold nanorods were prepared as described elsewhere[3] with some modification. Gold nanorods were synthesized by adding an aliquot of gold seeds (1 mL, 0.25 mM) under vigorous stirring to a growth solution comprising CTAB (100 mL, 100 mM), $HAuCl_4$ (1 mL, 0.05 mM), $AgNO_3$ (1.4 mL, 100 mM) and HQ (15 mL, 100 mM) at 30°C. The stirring was stopped after 5 min and the mixture was left for 6 h at 30°C. The nanoparticles were washed by two centrifugation rounds (7000 rpm, 30 min), to remove excess reagents and redispersed in CTAB (100 mM). The final gold concentration was 1 mM. Oxidative etching of gold nanorods (LSPR= 940 nm) was performed by means of the Au^{3+}-CTAB complex. To a solution containing gold nanorods (50 mL, 1 mM) in CTAB (100 mM), an aliquot of Au^{3+}-CTAB complex (5 mL, $[HAuCl_4]$ = 1 mM, [CTAB] = 100 mM) was added dropwise at 30°C under vigorous stirring for 30 min. Subsequently, the gold nanorods were centrifuged twice (7500 rpm, 30 min) and after the second centrifugation step, the nanoparticles were redispersed in CTAB (10 mM) to a final gold concentration of 0.4 mM.

Citrate Functionalization:[4] A suspension of gold nanorods (50 mL, 0.4 mM) in CTAB (10 mM) was subjected to three cycles of centrifugation (7500 rpm, 30 min) and redispersion

with Na-PSS (50 mL, 0.15 % wt) to remove the CTAB. The PSS-stabilized gold nanorods were centrifuged and redispersed in sodium citrate (50 mL, 5 mM) and the solution was incubated overnight for exchange PSS. Finally, the gold nanorods were subjected to two additional centrifugation and redispersion cycles using sodium citrate (50 mL, 5 mM), yielding stable dispersions of citrate-stabilized gold nanorods.

Ligand Exchange with DNA: DNA functionalization (15-mer) of AuNRs was carried out using the reported literature protocol.[5] In a typical experiment, citrate-coated AuNRs (500μL, 8.5 nM in terms of NPs) were combined with 10μL of DNA (100μM). The mixture was stored in a −20°C fridge for 2h. The ratio between [DNA] and [AuNRs] was ∼353. Subsequently, the mixture was then thawed at room temperature and purified by centrifugation at 5000 rpm for 10 minutes. The resulting pellet was redispersed in 100μL of 0.01% SDS solution. The centrifugation and redispersion process was repeated two more times.

S1.4 Experimental Setup

Figure S3 shows the optical photograph of the experimental setup. A four-window temperature-controlled cuvette holder (qpod-2e) was coupled with the laser beam and white light (spectrophotometer), both positioned orthogonal to monitor in real-time the change of optical properties of the dispersion. A continuous wave laser (808 nm) was used as the light source to carry out the photothermal redispersion of the AuNP assembly. The laser light was coupled to an optical fiber, connected in turn to one of the qpod-2e ports with a collimator lens (beam diameter = 5 mm). The light beam passing through the sample was collected in a beam stop. UV-Vis-NIR spectra were recorded by means of a spectrophotometer (MayaPro 2000, Ocean Optics) equipped with a tungsten/halogen light source, both connected with collimator lenses with 400 μm optical fibers. A micro-cuvette (optical path l = 1 cm), containing a binary mixture of AuSs and AuNRs (200 μL), was put in qpod-2e and encased within a Peltier element, so to achieve temperature control at all sites and at the bottom. UV-Vis-NIR spectra of dispersions were recorded every 2 seconds. The laser power and the

base temperature was changed on demand during the course of experiments, to get oscillations in the extinction of the dispersion. Optical extinction spectra, shown in Figure 2b were recorded using an Agilent Cary 3500 UV-visible spectrophotometer.

AuNP dispersion showing oscillations was prepared by adding 100 μL of DNA-coated AuSs (0.93mM in terms of Au^0) and 20 μL of DNA-coated AuNRs (2.98mM in terms of Au^0) to 100 μL RNA free BioPerformance certified water. 25 μL of 2M NaCl was added to the dispersion to increase the ionic strength of the solution. Finally, 7 μL of aggregating DNA (100 μM) was added to the dispersion to initiate the aggregation of AuNPs.

S1.5 Data and Code

All data and the code used to obtain the figures shown in the main text and the supporting information are available at https://github.com/anishrao/Pr003-self-oscillations. The code was written in Jupyter Notebook and the environment used during the course of the study is also provided in the project folder.

S1.6 Transmission Electron Microscopy (TEM) Studies

We performed TEM experiments in order to characterize the dimensions of AuSs, AuNRs, and their assemblies. Samples for TEM were prepared using 200 μL of AuNPs that was centrifuged and redispersed in 10 μL of Milli-Q water. Finally, 5 μL of the drop was placed on a formvar-coated Copper grid. The drop was allowed to slowly evaporate overnight at room temperature before imaging. TEM images were acquired with a FEI Tecnai G2 20 TWIN transmission electron microscope operating at an acceleration voltage of 200 kV.

S1.7 UV-Vis-NIR Studies:

Optical extinction spectra shown in Figures 2b, 2c S1, S2, S4, S6 were recorded with an Agilent Cary 3500 UV-visible spectrophotometer. All other spectra showing real-time mon-

itoring of the UV-Vis-NIR response of the system was recorded using MayaPro 2000 from Ocean Optics.

S2 UV-Vis-NIR of plasmonic NPs

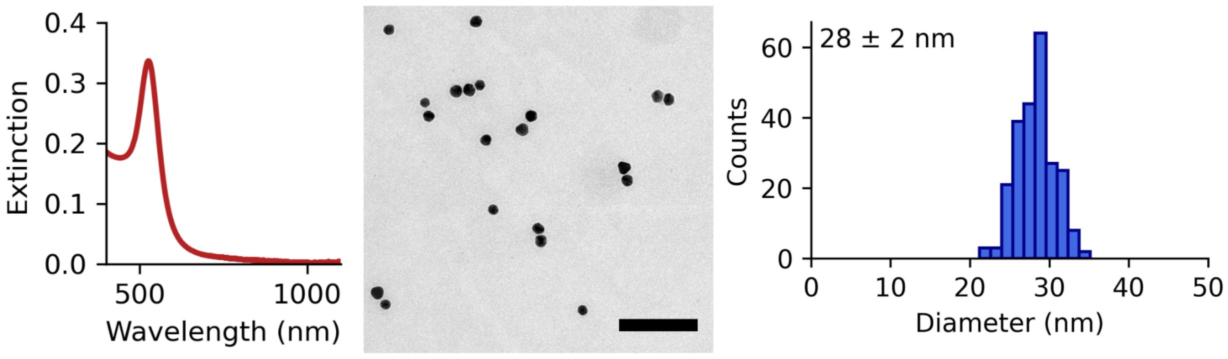

Figure S1: a) Extinction spectra and b) TEM image and accompanying size distribution histogram of AuSs. Scale bar = 200nm.

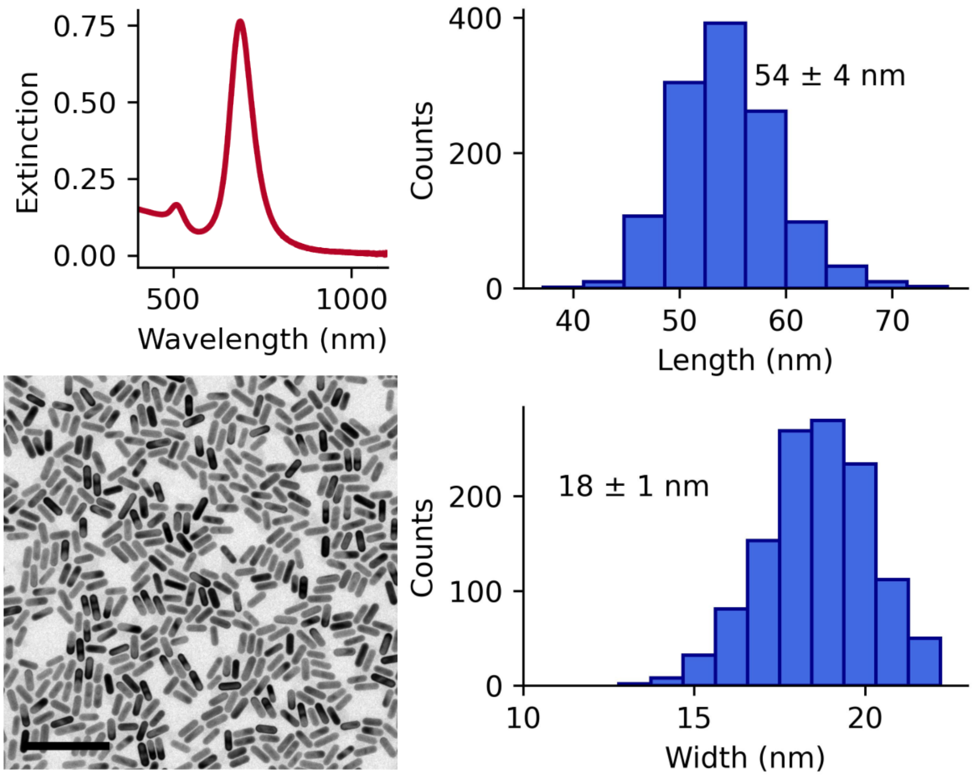

Figure S2: a) Extinction spectra and b) TEM image and accompanying size distribution histogram of AuNRs. Scale bar = 200nm.

S3 Experimental Setup

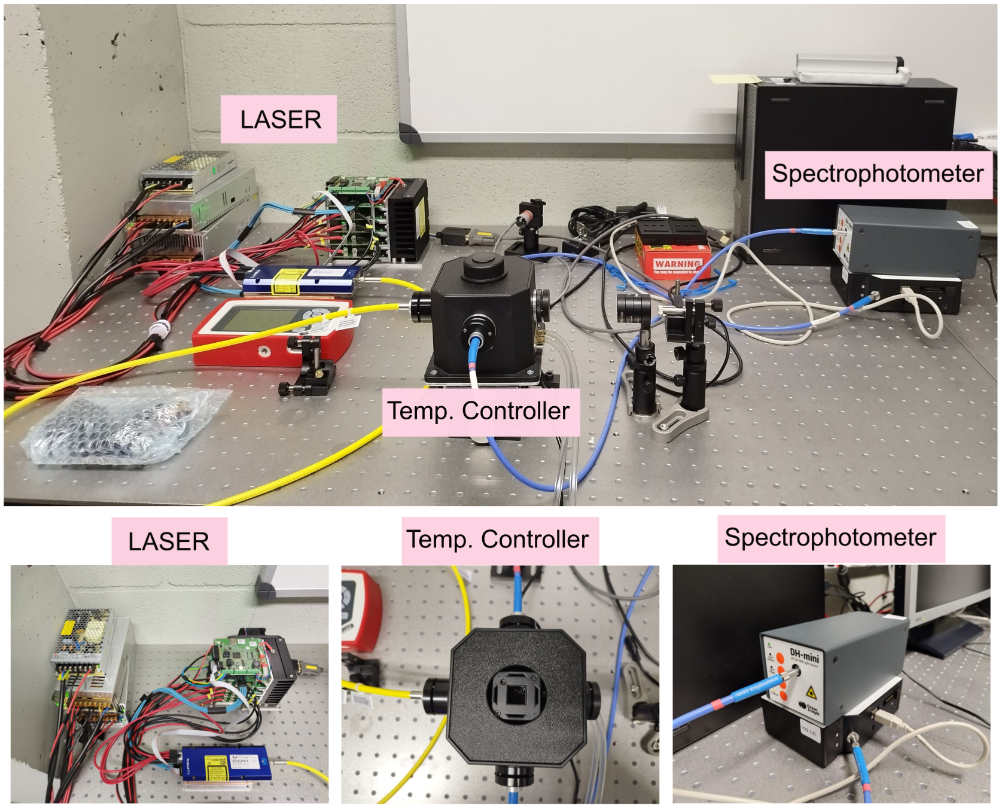

Figure S3: Optical photograph showing the complete experimental setup used for light irradiation and oscillation studies.

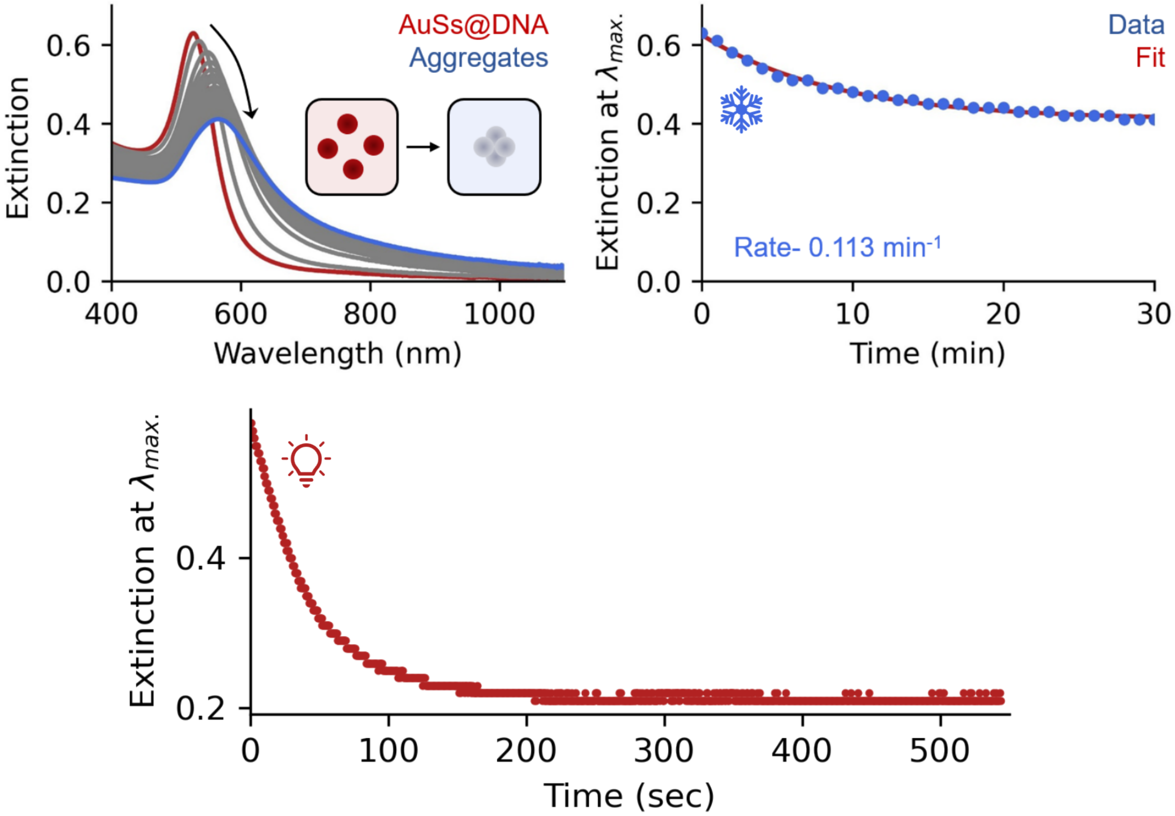

Figure S4: Time-dependent UV-Vis-NIR spectra showing the variation in the extinction of the AuSs@DNA in the presence of aggregating DNA. Here, the system shows a consistent redshift and a simultaneous decrease in the extinction intensity due to non-specific plasmon coupling. Panel b) shows the decrease in the extinction intensity at $\lambda_{max.}$ during the course of aggregation. Panel c) shows the inability of 808 nm laser even at 1.9 W to stop the disassembly of AuSs due to photothermal heating. This clearly shows the poor photothermal heating ability of AuSs.

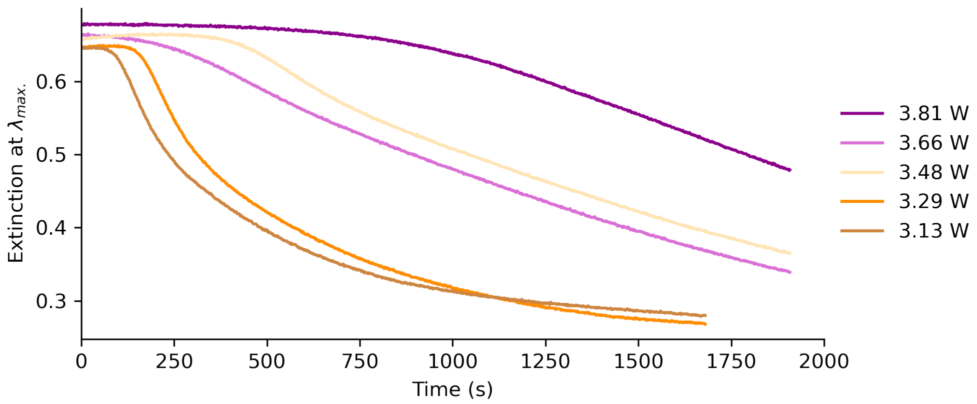

Figure S5: Figure showing the variation of extinction at $\lambda_{max.}$ during the course of aggregation at different laser powers. Note that here 670 nm CW laser was used for illumination. Here, 670 nm illumination is absorbed more by AuSs than 808 nm illumination. Even with this higher absorption, AuSs fail to retard the aggregation of AuSs even at ∼3.8 W.

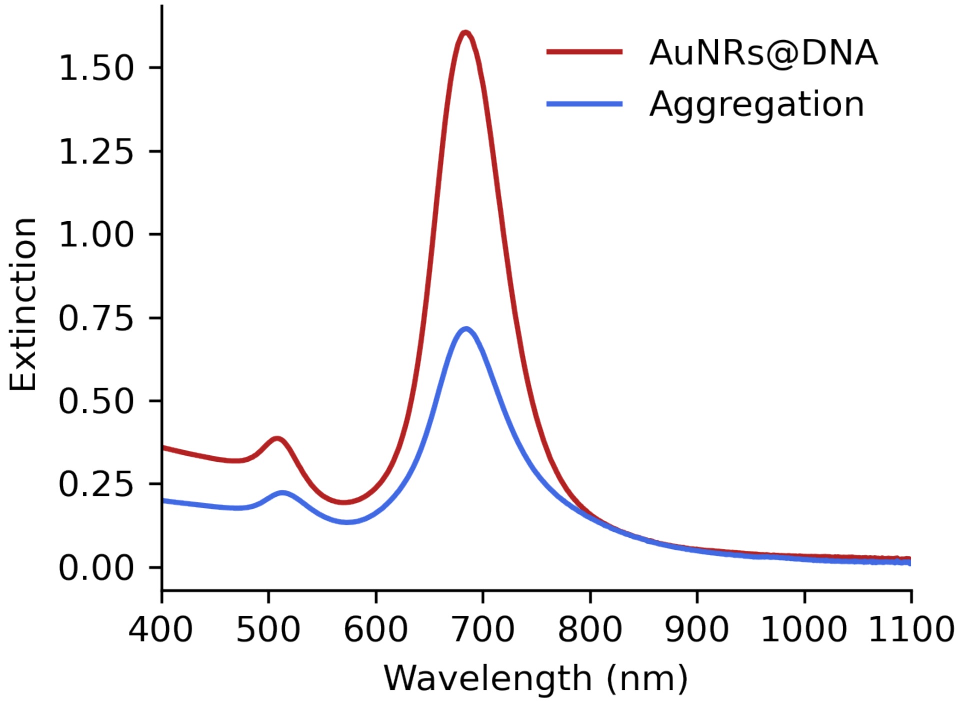

Figure S6: Figure showing the extinction spectrum of AuNRs coated with DNA before (shown in red) and after (shown in blue) the addition of aggregating DNA. Note that we observe minimal redshifts upon the addition of AuNRs.

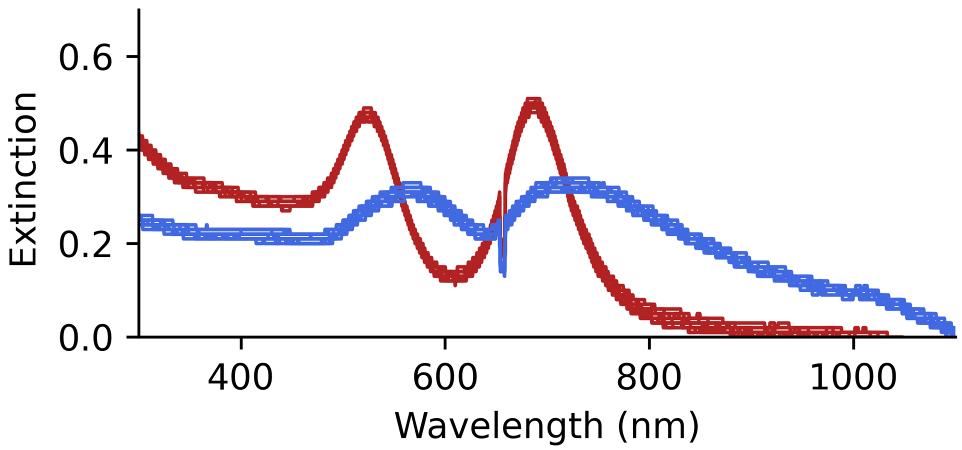

Figure S7: Figure showing the extinction spectrum of binary mixture of AuSs and AuNRs during the reversibility cycles. Here, blue and red curves indicate assembled and disassembled samples, respectively.

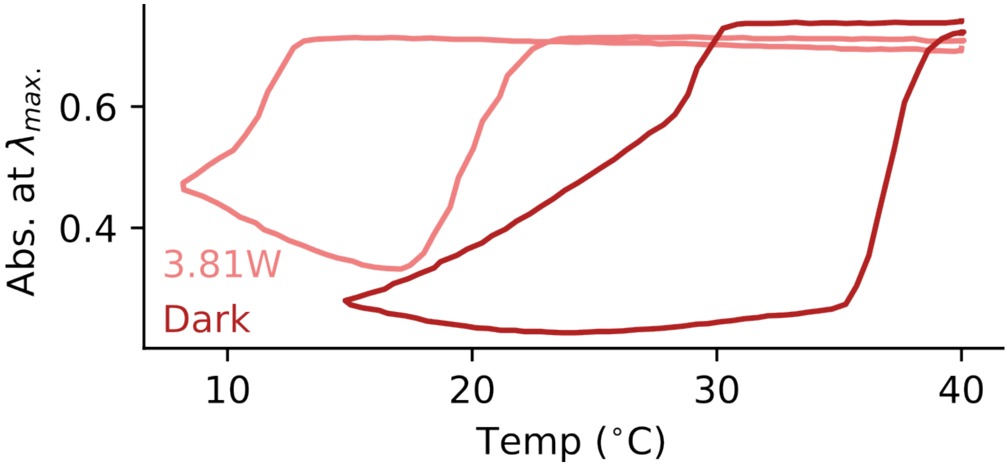

Figure S8: Variation in the hysteresis of DNA-coated AuSs in the presence (pink) and absence (red) of light (CW 670 nm laser). Here, thermal hysteresis of the system was followed using extinction at $\lambda_{max.}$ within a temperature range spanning 5°C to 40°C, at a scan rate of 0.5°C/min. It is worth noting that the melting and freezing temperature decreases from 37 to 29 °C and 19 to 12 °C, respectively. Note that the experiment was performed with a sample 1mL in volume as opposed to 200 μL for the binary AuNP mixture.

S4 Mechanism

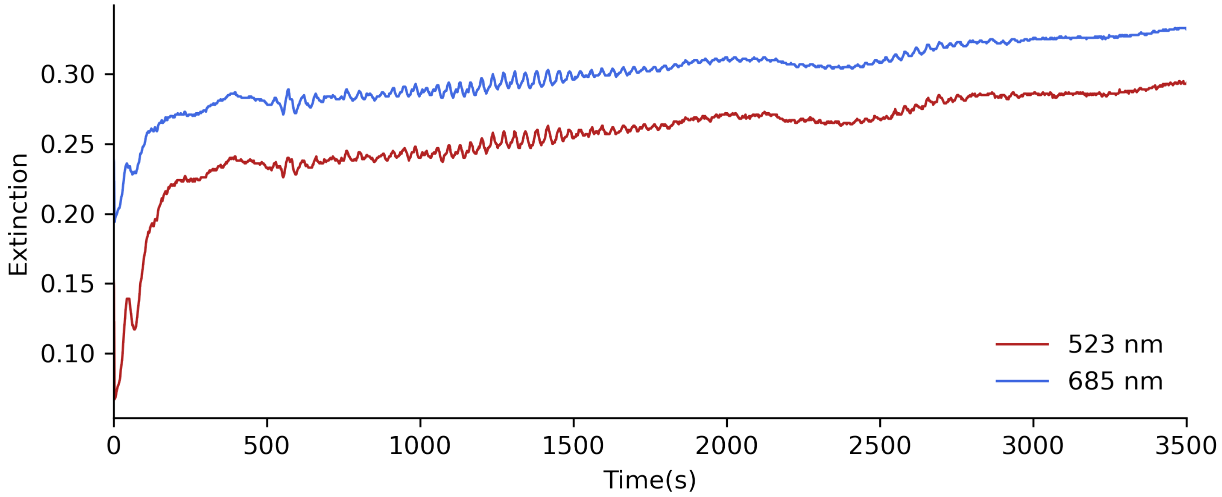

Figure S9: Figure showing the oscillating extinction response of the system at 15°C and a laser power of ∼135 mW at both 685 nm (corresponding to AuNRs) and 523 nm (corresponding to AuSs).

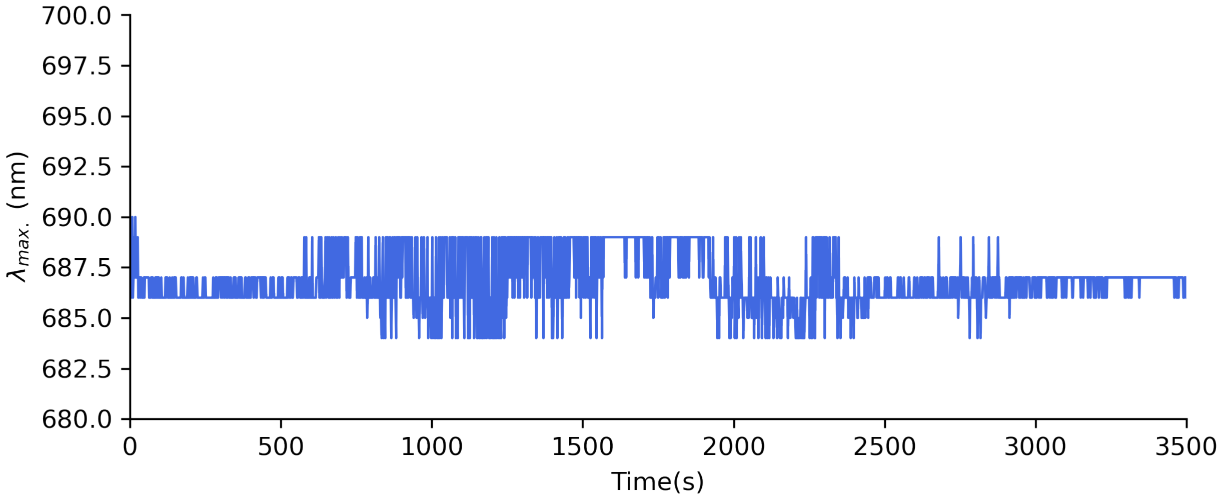

Figure S10: Figure showing the variations in $\lambda_{max.}$ of the system at 15°C and a laser power of ∼135 mW.

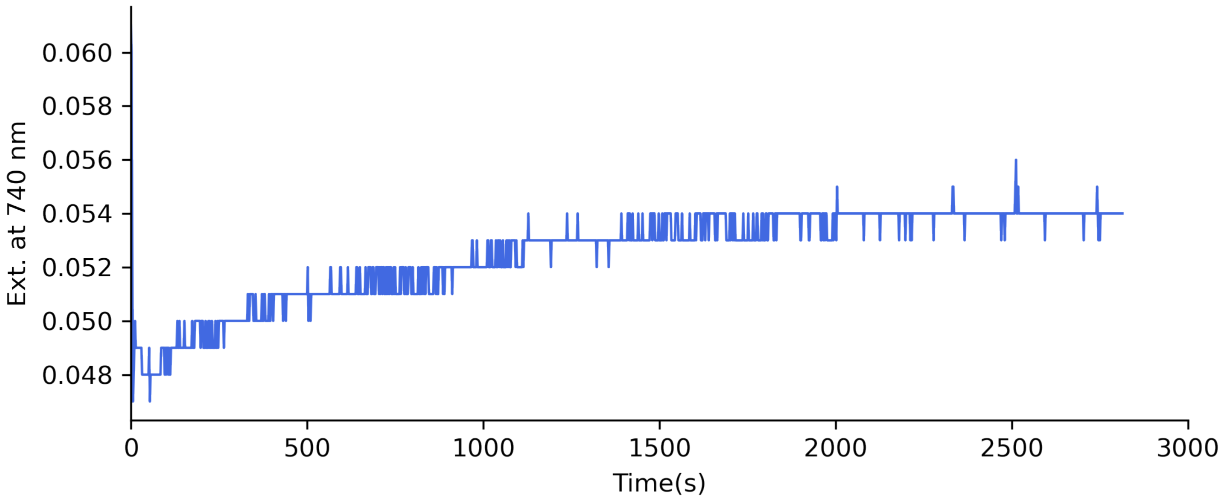

Figure S11: Figure showing the variations in extinction of CTAB coated AuNRs (LSPR at 808 nm), at 15°C and a laser power of ∼135 mW.

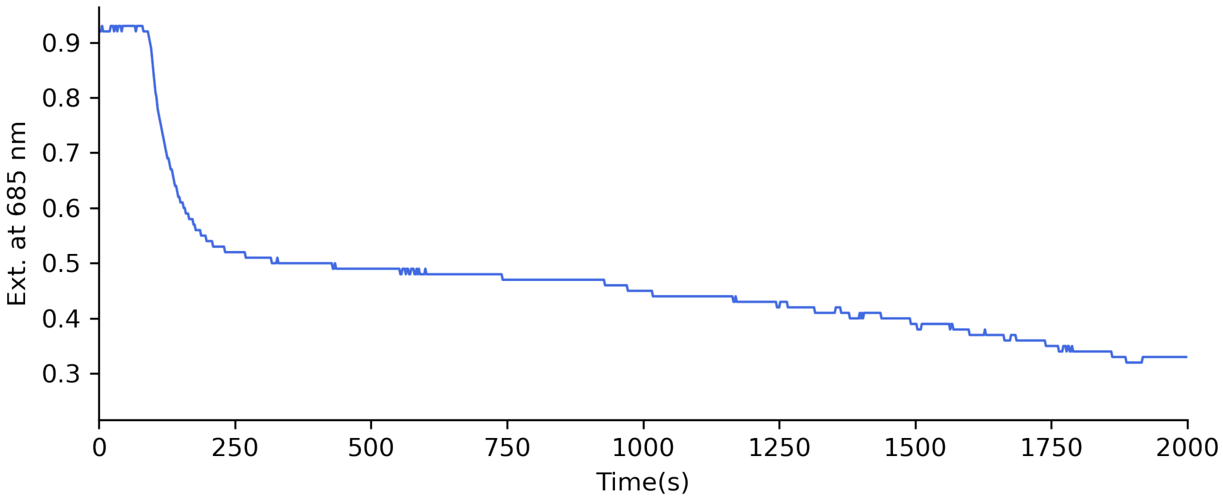

Figure S12: Figure showing the variations in Extinction of binary mixture of AuNPs, at 15°C and a laser power of ∼135 mW. Notably, in this case, the system was **not** allowed to aggregate before initiating the oscillation experiment. Note that other than the initial state (unassembled NPs), every other experimental parameter was unchanged. Additionally, we observe that when exposed to ∼135 mW at 15°C for longer irradiation time, the AuNP mixture indeed shows aggregation. In contrast to the lack of hysteresis under these conditions is possibly due to the temperature ramp, where, the system does not get sufficient time to aggregate under low temperature conditions.

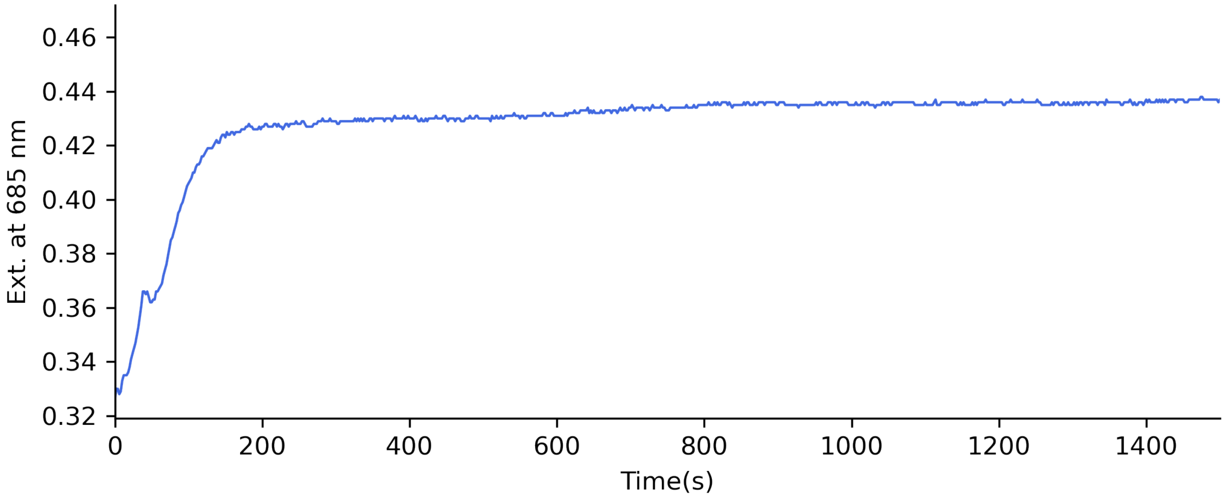

Figure S13: Figure showing the variations in Extinction of binary mixture of AuNPs, at 15°C and a laser power of ∼135 mW. Notably, in this case, the system was allowed to aggregate for 30 mins. after the addition of aggregating DNA, as opposed to complete precipitation in instances where the system showed oscillations. Note that other than the initial state (partial aggregates), every other experimental parameter was unchanged.

References

(1) Bastús, N. G.; Comenge, J.; Puntes, V. Kinetically controlled seeded growth synthesis of citrate-stabilized gold nanoparticles of up to 200 nm: size focusing versus Ostwald ripening. *Langmuir* **2011**, *27*, 11098–11105.

(2) Hurst, S. J.; Lytton-Jean, A. K.; Mirkin, C. A. Maximizing DNA loading on a range of gold nanoparticle sizes. *Anal. Chem.* **2006**, *78*, 8313–8318.

(3) Vigderman, L.; Zubarev, E. R. High-yield synthesis of gold nanorods with longitudinal SPR peak greater than 1200 nm using hydroquinone as a reducing agent. *Chem. Mater.* **2013**, *25*, 1450–1457.

(4) Mehtala, J. G.; Zemlyanov, D. Y.; Max, J. P.; Kadasala, N.; Zhao, S.; Wei, A. Citrate-stabilized gold nanorods. *Langmuir* **2014**, *30*, 13727–13730.

(5) Liu, B.; Liu, J. Freezing directed construction of bio/nano interfaces: reagentless conjugation, denser spherical nucleic acids, and better nanoflares. *J. Am. Chem. Soc.* **2017**, *139*, 9471–9474.
